# Circ_0000285 regulates proliferation, migration, invasion and apoptosis of osteosarcoma by miR-409-3p/IGFBP3 axis

**DOI:** 10.1186/s12935-020-01557-5

**Published:** 2020-10-06

**Authors:** Zhisheng Long, Feipeng Gong, Yuxu Li, Zhiqiang Fan, Jingtang Li

**Affiliations:** Department of Orthopaedics, Jiangxi Provincial People’s Hospital Affiliated to Nanchang University, No.92, AiGuo Road, Nanchang City, 330006 Jiangxi Province China

**Keywords:** circ_0000285, miR-409-3p, IGFBP3, Cell growth, OS

## Abstract

**Background:**

Circular RNAs (circRNAs) are important regulators in the pathogenesis of diseases and affects the occurrence and development of diseases. However, the role of circRNAs in osteosarcoma (OS) has not been fully elucidated.

**Methods:**

The expression of circ_0000285, miR-409-3p and insulin-like growth factor binding protein 3 (IGFBP3) was detected using quantitative real-time PCR (qRT-PCR). The protein level of IGFBP3 was measured using western blot. CCK-8 and colony formation assays were used to determine cell proliferation. Flow cytometry was applied to measure cell cycle and cell apoptosis. Transwell assay was used to assess cell invasion and migration. Dual-luciferase reporter assay and RNA Binding Protein Immunoprecipitation (RIP) assay were performed to determine the relationship among circ_0000285, miR-409-3p and IGFBP3. The animal experiments were performed to determine the function of circ_0000285 in vivo.

**Results:**

In this study, we found that the expression of circ_0000285 was significantly increased in OS tissues and cells and was enriched in the cytoplasm. Knockdown of circ_0000285 inhibited OS growth in vitro and in vivo. Moreover, miR-409-3p was a target miRNA of circ_0000285 and miR-409-3p targets to IGFBP3 in OS. Besides, circ_0000285 could promote proliferation, migration, invasion and inhibit apoptosis of osteosarcoma by miR-409-3p/IGFBP3 axis.

**Conclusion:**

In this study, circ_0000285 regulated proliferation, migration, invasion and apoptosis of OS cells by miR-409-3p/IGFBP3 axis, implying that circ_0000285 was a potential target for OS therapy.

## Highlights

1. circ_0000285 expression was enhanced in osteosarcoma tissues and cells.
2. Suppression of circ_0000285 inhibited osteosarcoma progression in vitro and in vivo.
3. circ_0000285 targeting miR-409-3p to upregulate IGFBP3, and consequently regulates proliferation, migration, invasion and apoptosis of osteosarcoma.

## Background

Osteosarcoma (OS) is a type of malignant tumor with a high incidence in adolescents and about 60% of OS patients are under 25 years of age [1–3]. The cause and mechanism of osteosarcoma are still unclear, which make it difficult to diagnose and treat osteosarcoma.

Cirular RNA (circRNA) is a special type of non-coding RNA with closed-loop structure and its expression is stable in cells [4–6]. Besides, circRNAs were related to tumor development and were biomarkers for cancer, including hepatocellular carcinoma (HCC), non-small cell lung cancer, colorectal cancer, breast cancer and OS [6–11]. For example, circRNA MTO1 was down-regulated in breast cancer and HCC [12, 13]. Moreover, the promotion of circRNA MTO1 suppressed cell viability, promoted cell cytotoxicity and reversed monastrol resistance in breast cancer [13]. Recent research demonstrates that circ_0000285 acts as an oncogene in laryngocarcinoma [14]. In hepatocellular carcinoma, circ_0000285 were found enhance cell metastasis of HCC by targeting miR-599 and might be a potential therapeutic target [15]. In this study, we found that circ_0000285 was up-regulated in OS and predicted that circ_0000285 might play a certain role in OS occurrence. However, the role of circ_0000285 has not been fully elucidated in OS.

More than that, microRNA (miRNA)-rich binding sites on circRNA molecules regulate the expression of target genes in cells by binding miRNAs in various cancers [16, 17]. Chen et al. determined that circ_100290 affected oral cancer proliferation via sponging miR-29 family [18]. Otherwise, circRNA_0008717, acted as an oncogene, was up-regulated in OS and play a role through targeting miR-203 in OS [19]. Moreover, circ_0067934 regulated HCC tumor growth through targeting miR-1324/FZD5 axis [20]. However, the regulatory mechanism of circ_0000285 in OS has not been explored.

In this paper, we found that circ_0000285 was highly expressed in OS cells, and it is speculated that circ_0000285 might be an important regulator in the development of OS. Using modern bioinformatics technology, we have demonstrated that circ_0000285 regulated insulin-like growth factor binding protein 3 (IGFBP3) expression in OS by binding to miR-409-3p. Therefore, we predicted that circ_0000285/miR-409-3p/IGFBP3 axis played an indispensable role in the cell development of OS.

## Materials and methods

### Patients and tissues

30 pairs of OS and adjacent tissues were collected from patients who have been diagnosed with OS in Jiangxi Provincial People's Hospital Affiliated to Nanchang University. This experiment was approved by the ethics committee of jiangxi Provincial People's Hospital Affiliated to Nanchang University and the patient's written consent has been obtained. The clinicopathologic features of these patients were presented in Additional file 1. Table S1. All samples were taken in liquid nitrogen and stored in − 80 °C refrigerator for subsequent experiments.

### Cell culture and cell transfection

The human osteoblasts (hFOB 1.19) and OS cells (SJSA1 and U2OS) were purchased from the Cell Bank of Type Culture Collection of Chinese Academy of Science. All cells were maintained in RPMI-1640 medium (catalog number: 22400071, Gibco, Carlsbad, CA, USA) supplemented with 10% FBS at 37 °C in a humid environment contained 5% CO_2_.

si-circ_0000285, si-negative control (NC), miR-409-3p inhibitor, inhibitor NC, miR-409-3p mimic, miR-NC mimic (miRNA NC), LVX-circ_0000285 (circ_0000285), pLVX-Puro lentivirus empty vector (pLVX), pcDNA3.1-IGFBP3 (pc-IGFBP3) and pcDNA3.1 empty vector were purchased from GenePharma (Shanghai, China). When the cells were fused to 60–80%, the vectors and oligonucleotides were transfected into SJSA1 and U2OS using Lipofectamine 2000 reagent (catalog number: 11668019; Invitrogen, Carlsbad, Calif, USA).

### Quantitative Real Time PCR (qRT-PCR) and RNase R assay

RNA of tissues and cells was extracted using TRIzol Reagent (catalog number: 15596018, Invitrogen). RNase R (3 U/mg, Epicentre Biotechnologies, Madison, WI, USA) was used to treat RNA for 15 min at 37 °C for RNase R treatment. Then the NanoDrop 2000 was used to detect the RNA quality. A TaqMan™ microRNA assay kit (catalog number: RNR07250, Applied Biosystems, Foster City, CA, USA) was used to detect the expression of miR-409-3p. Prime Script RT Master Mix (catalog number: RR036B, Takara, Tokyo, Japan) was applied to detect the expression of circ_0000285, IGFBP3 and HIPK3. Then SYBR-Green PCR kit (catalog number: 204143, QIAGEN, Hilden, Germany) was applied to reaction on an ABI 7300 Fast Real-Time PCR system. U6 or GAPDH were presented as internal genes of miR-409-3p or IGFBP3, HIPK3 and circ_0000285, respectively.

Primer sequences were as follows:

miR-409-3p Forward 5′-GAATGTTGCTCGGTGA-3′ and reverse 5′-GTGCAGGGTCCGAGGT-3’.

circ_0000285 Forward 5′-TACCTCTGCAGGCAGGAACT-3′ and reverse 5′-TCACATGAATTTAGGTGGGACTT-3’.

IGFBP3 Forward 5′-ATAATCATCATCAAGAAAGGGCA-3′ and reverse 5′-AGTTCTGGGTATCTGTGCTCTGA-3’.

HIPK3 Forward 5′-ACATTGGAAGAGCATGAGGCAGAGA-3′ and reverse 5′-CTGCTGAAAAGCATCACCACAACCA-3’.

GAPDH Forward 5ʹ-GCCATCACAGCAACACAGAA-3ʹ and reverse 5ʹ-GCCATACCAGTAAGCTTG CC-3ʹ.

U6 Forward 5ʹ-CGCTTCGGCAGCACATATAC-3ʹ and reverse 5ʹ-TTCACGAATTTGCGTGTCAT-3ʹ.

### Actinomycin D assay

Transfected cells were seeded in 24-well plates and cultured for 24 h. Then cells were treated with Actinomycin D for 24 h. After that, the expression of circ_0000285 was detected using qRT-PCR.

### Cytoplasmic and Nuclear RNA isolation

Cytoplasmic and Nuclear RNA was separated from total RNA using a PARIS kit (catalog number: AM1921, Invitrogen, Carlsbad, CA, USA) following the instructions. U6 and GAPDH were circ_0000285 nuclear internal and external control, respectively.

### Western blot

Total protein was separated by sodium dodecyl sulfate–polyacrylamide gel electrophoresis (SDS-PAGE) with 120 V and then transferred onto nitrocellulose membranes. After the membranes were blocked with non-fat milk, the membranes were incubated with primary antibodies PARP (46D11) Rabbit mAb, IGFBP3 (D1U9C) Rabbit mAb and GAPDH (D16H11) XP® Rabbit mAb (catalog number: 9532; 25864; 5174, Cell Signaling Technology, Danvers, MA, USA) overnight at 4 °C. Next, the membranes were incubated with anti-rabbit IgG, HRP-linked antibody (catalog number: 7074; Cell Signaling Technology). Finally, the blot was detected and analyzed by using Image-Pro Plus software (Mediacy, Inc., Rockville, MD, USA).

### Cell counting kit-8 (CCK-8)

CCK-8 kit (catalog number: Dojindo, Shanghai, China) was used to detect cell proliferation. Transfected cells were maintained in 96-well plates and the CCK-8 solution was put into each well for 2 h at room temperature. Finally, cell proliferation was analyzed using SpectraMax M5 microplate reader (Molecular Devices, LLC, Sunnyvale, CA, USA) at 450 nm.

### Transwell migration and invasion

Cells were seeded and cultured in Transwell upper chamber (catalog number: ECM509, 8 mm pores, Millipore, Billerica, MA, USA). The chamber with matrigel (catalog number: 354234, BD Biosciences, Franklin Lakes, NJ, USA) was used to measure cell invasion, while the chamber without matrigel was used to measure cell migration. RPMI-1640 medium was added into the lower chamber and incubated with cells for 24 h. Then, the cells in the lower chamber were stained with the crystal violet. Finally, cells were counted and viewed with a microscope.

### Colony formation assay

Transfected cells were seeded in a six-well plate and incubated in RPMI-1640 medium (catalog number: 22400071, Gibco) for 14 days. Then the crystal violet was added into each well to stain cells and the numbers of the colony were counted in a microscope.

### Flow cytometry

The PI/Annexin V-FITC Apoptosis Detection Kit (catalog number: 556547, BD Biosciences) was applied to measure cell apoptosis. Briefly, transfected cells were seeded in 6-well plates. Annexin V-FITC and PI were added into wells and the apoptotic cells were measured using flow cytometer (BD Biosciences).

Cell cycle was detected using Cycletest™ Plus DNA Reagent kit (catalog number: 340242, BD Biosciences). Briefly, transfected cells were labeled with PI solution before treated with RNAase for a half hour in darkness and cell cycle were measured using flow cytometer (BD Biosciences).

### Dual-luciferase reporter assay

Circ_0000285 or IGFBP3 wild type sequences (contained the binding sites of miR-409-3p) and circ_0000285 or IGFBP3 mutate sequences (contained the mutate sites) were synthesized and inserted into pmirGLO vectors (Promega, Madison, WI, USA) to construct experimental vectors named WT-circ_0000285, WT-IGFBP3-3′UTR, MUT-circ_0000285, MUT-IGFBP3-3′UTR. Then, these vectors were separately co-transfected with miR-409-3p mimic or miRNA NC into SJSA1 and U2OS cells using Lipofectamine 2000 reagent (catalog number: 11668019; Invitrogen). 48 h after transfection, luciferase activities were measured using the Dual-Luciferase Reporter Assay System (catalog number: E1910, Promega).

### RNA immunoprecipitation protocol (RIP) assay

RIP assay was performed by a Magna RIP ™ RNA-Binding Protein Immunoprecipitation Kit (catalog number: 17-701, Merck Life Science, Shanghai, China). Transfected cells were lysed in cold RIP assay buffer and incubated with antibodies anti-Ago2 or anti-IgG (catalog number: 2897; 14708, Cell Signaling Technology). Then, RNA was extracted and the enrichment of circ_0000285, miR-409-3p and IGFBP3 was determined by qRT-PCR.

### Animal experiments

Female BALB/c nude mice (4–5 weeks old) were purchased from the Shanghai Institute for Biological Sciences (SIBS, Shanghai, China). U2OS cells transfected with sh-circ_0000285 or sh-NC were subcutaneously injected into flank sides of mice. Tumor width and length were measured every week. After transfection for 28 days, the mice were sacrificed and tumor weight was measured. This study was approved by the animal ethics committee of iangxi Provincial People's Hospital Affiliated to Nanchang University.

### Statistical analysis

All data were analyzed and performed using GraphPad Prism software (GraphPad, San Diego, CA, USA) and presented as mean ± standard deviation (SD). The statistical significance of the groups was analyzed using Student’s t-test or one-way analysis of variance. *P* value < 0.05 was employed as statistically significant.

## Results

### Circ_0000285 expression was upregulated in OS

**Fig. 1:**
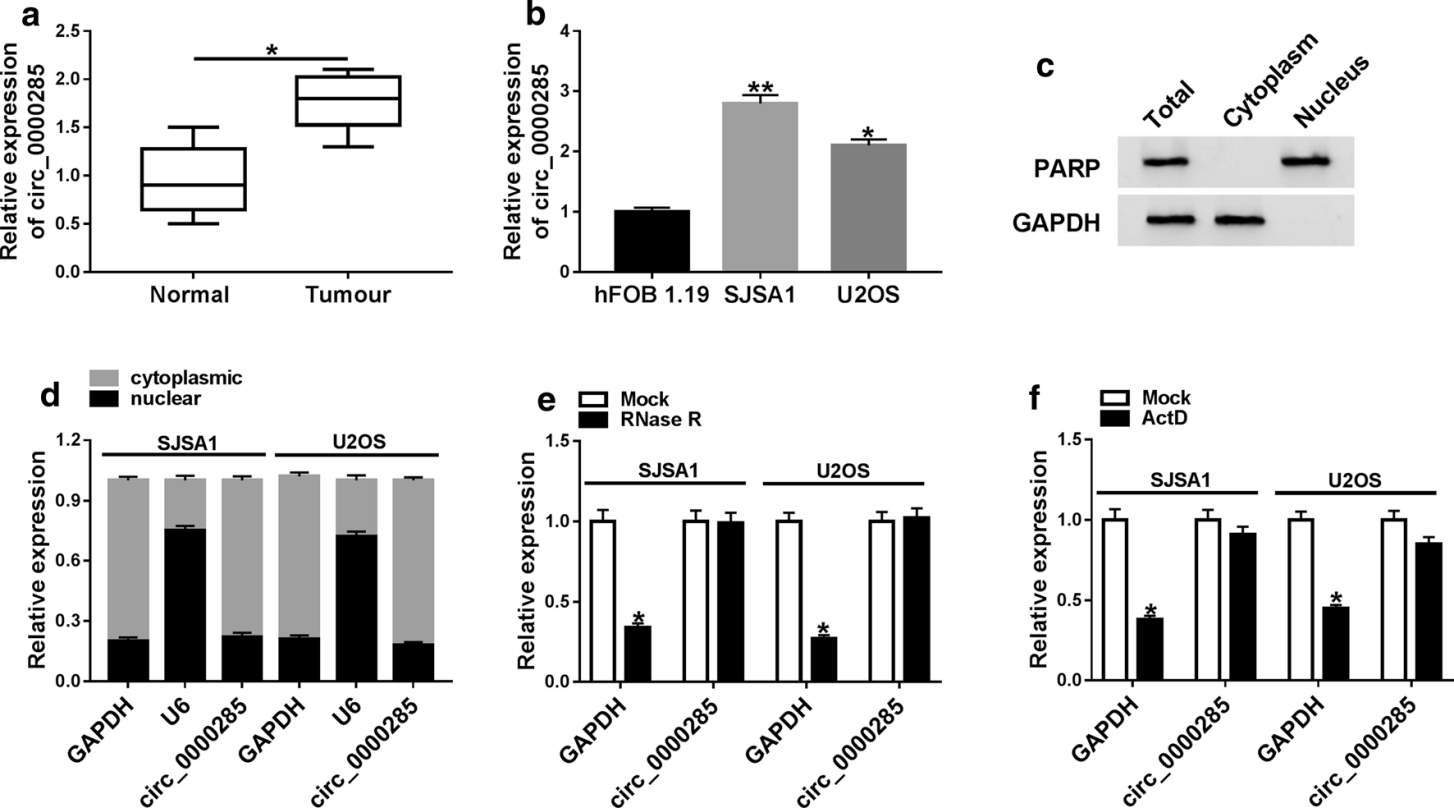
Circ_0000285 expression was upregulated in OS. **a** QRT-PCR was used to detect the expression of circ_0000285 in normal tissues and tumor tissues. **b** QRT-PCR was used to detect the expression of circ_0000285 in normal cells (hFOB 1.19) and tumor cells (SJSA1 and U2OS). **c** Western blot was used to detect whether PARP and GAPDH protein were in total (protein), Cytoplasm and Nucleus. **d** qRT-PCR determined that circ_0000285 expressed high in cytoplasm in SJSA1 and U2OS cells. **e**, **f** qRT-PCR was used to detect the expression of circ_0000285 with RNase R and Actinomycin D treated RNA in SJSA1 and U2OS cells. *P < 0.05, **P < 0.01

To explore the function of circ_0000285 in OS progression and development, qRT-PCR was used to detect the expression of circ_0000285 in OS tissues and cells. Compared with normal tissues, circ_0000285 expression was significantly increased in OS tissues (Fig. 1a). Similarly, circ_0000285 expression of OS cells (SJSA1 and U2OS) was remarkably higher than that of normal cells (hFOB 1.19) (Fig. 1b). Additional file 1. Table S1 shows the clinicopathologic features of patients. The expression of circ_0000285 was positively associated with tumor size, however, the cancer differentiation grade seems unrelated to circ_0000285 expression. Furthermore, a PARIS kit (Life Technologies) was used to isolate cytoplasmic RNAs (circ_0000285 and U6), nuclear RNAs (circ_0000285 and U6), and total RNA as well as the PARP and GAPDH protein in cytoplasmic, nuclear and total proteins in OS tissues and normal tissues, the PARIS was a marker protein of the nucleus. The results of western blot showed that the GAPDH protein only existed in the cytoplasm, while the PARP protein only existed in the nucleus, indicating the success of the nucleo-cytoplasmic separation experiment (Fig. 1c). Following experiments determined circ_0000285 was mainly expressed in the cytoplasm of SJSA1 and U2OS cells (Fig. 1d). RNase R treatment determined that circ_0000285 was resistant to RNase R (Fig. 1e). Furthermore, Actinomycin D treatment showed that circ_0000285 was highly stable in SJSA1 and U2OS cells (Fig. 1f). Thus, these results suggested that circ_0000285 was associated with OS development and played a role in OS.

### Downregulation of circ_0000285 suppressed cell progression in OS

**Fig. 2:**
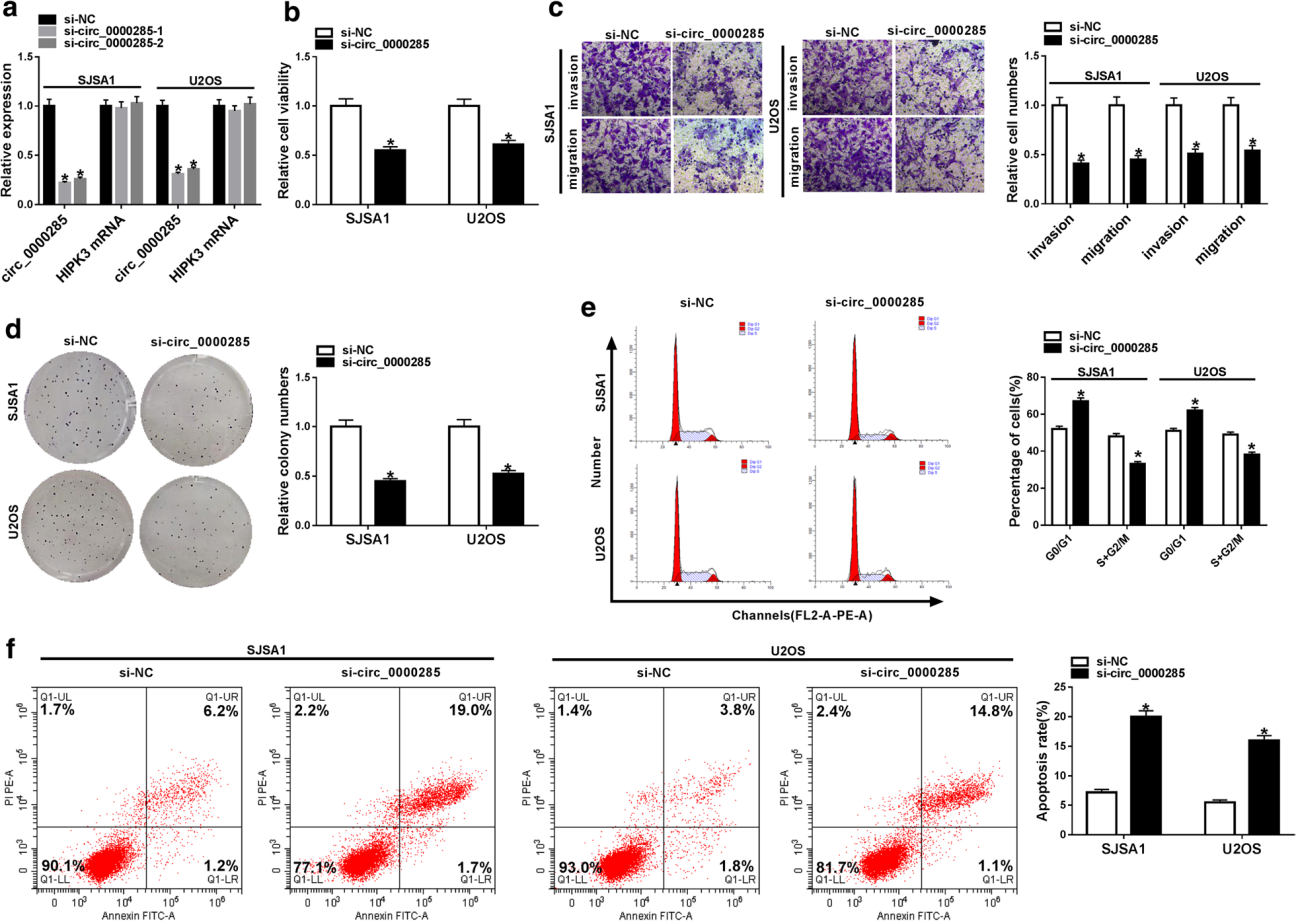
Downregulation of circ_0000285 suppressed cell progression in OS. **a** The expression of circ_0000285 and HIPK3 was measured in si-NC, si-circ_0000285-1 and si-circ_0000285-2 groups. **b** CCK-8 assay was used to detect cell viability in si-NC and si-circ_0000285 groups in SJSA1 and U2OS cells. **c** Transwell migration and invasion assays were used to assess cell migration and invasion in si-NC and si-circ_0000285 groups in SJSA1 and U2OS cells. **d** Colony formation assay was applied to assess cell proliferation in si-NC and si-circ_0000285 groups in SJSA1 and U2OS cells. **e**, **f** Flow cytometry was used to detect cell cycle and cell apoptosis in si-NC and si-circ_0000285 groups in SJSA1 and U2OS cells. *P < 0.05

As shown in Fig. 2a, we obtained two stable circ_0000285 low-expressing cell lines (si-circ_0000285-1 and si-circ_0000285-2) in SJSA1 and U2OS cells. qRT-PCR was applied to detect the expression of circ_0000285 and HIPK3 (the linear mRNA of circ_0000285) and the results showed that si-circ_0000285 transfection decreased circ_0000285 expression and had no effect on HIPK3 in SJSA1 and U2OS cells (Fig. 2a). CCK-8 assay and colony formation assay determined that knockdown of circ_0000285 could inhibit the capacity of cell proliferation in SJSA1 and U2OS cells (Fig. 2b and d). Also, transwell migration and invasion assays were used to measure the abilities of invasion and migration in OS, and the results showed that inhibition of circ_0000285 significantly reduced the abilities of invasion and migration in SJSA1 and U2OS cells (Fig. 2c). Moreover, si-circ_0000285 transfection could improve the percentage of cells at G0/G1 while decreased the percentage of cells at S + G2/M in SJSA1 and U2OS cells (Fig. 2e). Otherwise, the analysis of flow cytometry suggested that cell apoptosis was induced by downregulating circ_0000285 in SJSA1 and U2OS cells (Fig. 2f). Therefore, inhibition of circ_0000285 could suppress cell progression in OS.

### Circ_0000285 sponged miR-409-3p and IGFBP3 was a direct target of miR-409-3p in OS

**Fig. 3:**
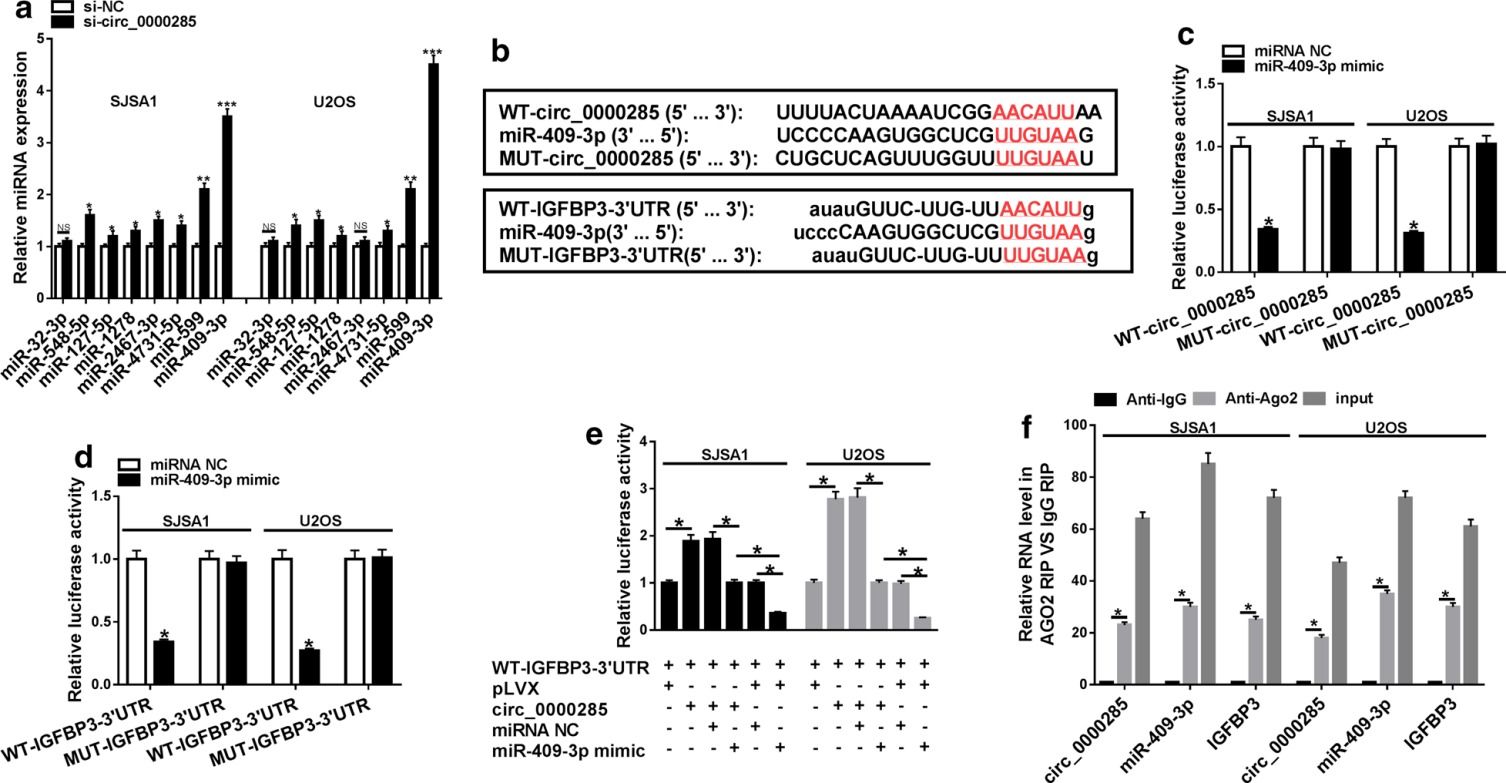
Circ_0000285 sponged miR-409-3p to target IGFBP3 in OS. **a** qRT-PCR was used to detect the expression of miR-32-3p, miR-548-5p, miR-127-5p, miR-1278, miR-2467-3p, miR-4731-5p, miR-599 and miR-409-3p in si-NC and si-circ_0000285 groups in SJSA1 and U2OS cells. **b** The predicted binding sites of miR-409-3p to the circ_0000285 and IGFBP3 sequences were shown. **c**, **d** Luciferase activities were detected in SJSA1 and U2OS cells co-transfected miR-409-3p mimic or miRNA NC with WT-circ_0000285 or MUT-circ_0000285 and WT-IGFBP3-3′UTR or MUT-IGFBP3-3′UTR. **e**, **f** RIP assay was applied to determine the relationship among circ_0000285, miR-409-3p and IGFBP3. *P < 0.05, **P < 0.01, ***P < 0.001

To further explore the regulatory mechanism of circ_0000285 in OS, circRNA intercomer and CircInteractome software were used to predict potential miRNAs of circ_0000285 and the results showed that 8 overlap miRNAs were predicted the two software, including miR-32-3p, miR-548-5p, miR-127-5p, miR-1278, miR-2467-3p, miR-4731-5p, miR-599 and miR-409-3p and their expression was detected in si-circ_0000285 and si-NC groups of SJSA1 and U2OS cells using qRT-PCR. The results showed the most significant increase in miR-409-3p expression (Fig. 3a). Besides, IGFBP3 had been predicted to be a potential mRNA of miR-409-3p and we also found circ_0000285 or IGFBP3-3′UTR had binding sites of miR-409-3p (Fig. 3b). The results of dual-luciferase reporter assay showed that luciferase activity was significantly reduced in SJSA1 and U2OS cells cotransfected with WT-circ_0000285 and miR-409-3p mimic (miR-409-3p) (Fig. 3c). Additionally, luciferase activity was significantly reduced in SJSA1 and U2OS cells cotransfected with WT-IGFBP3-3′UTR and miR-409-3p mimic (miR-409-3p) (Fig. 3d). Importantly, dual-luciferase reporter assay and RIP assay determined that circ_0000285 could regulate IGFBP3 through sponging miR-409-3p in SJSA1 and U2OS cells (Fig. 3e, f).

### Circ_0000285 upregulated IGFBP3 by sponging miR-409-3p

**Fig. 4:**
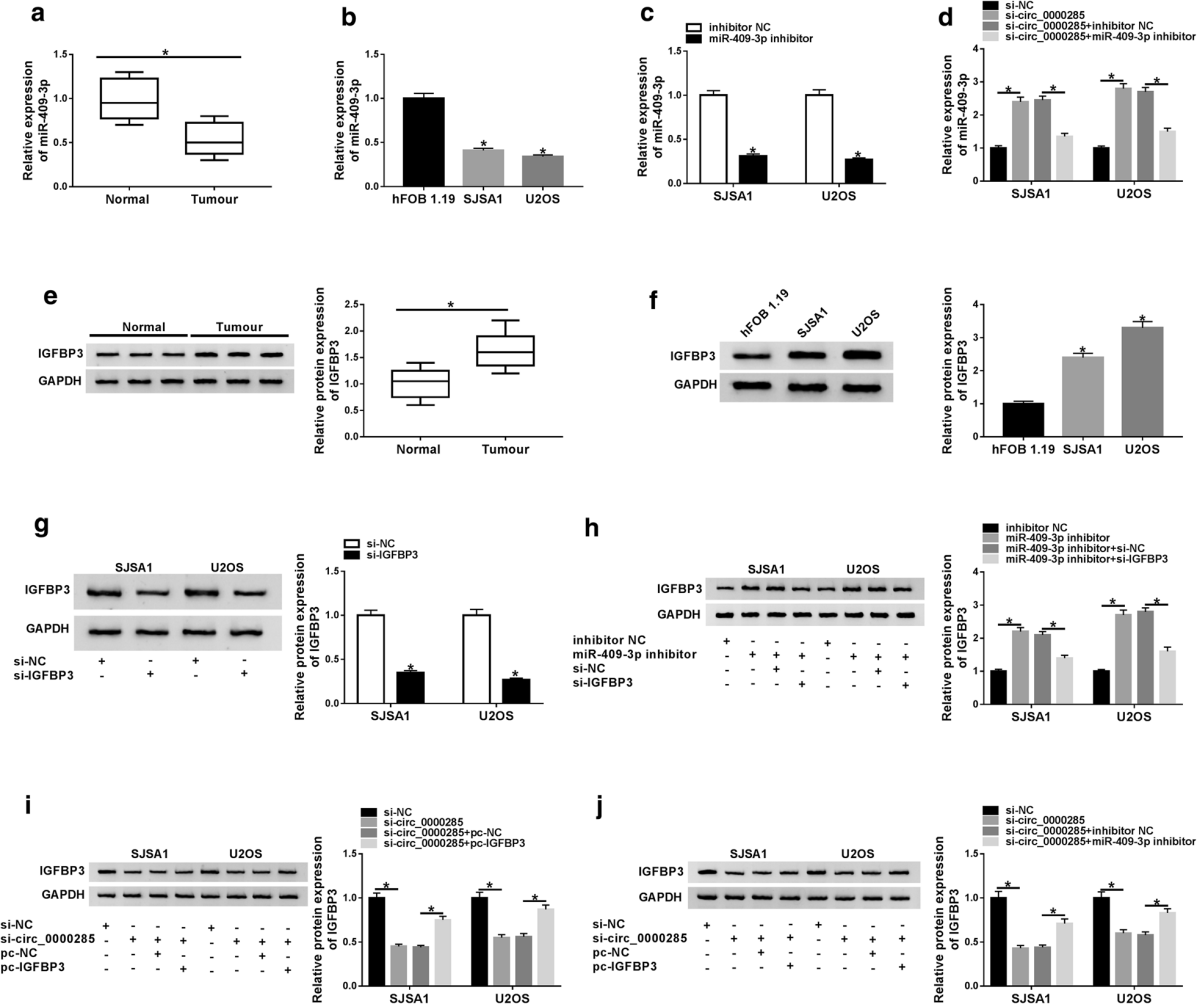
The expression of miR-409-3p was reduced while the expression of IGFBP3was increased in OS. **a** The expression of miR-409-3p was detected in normal tissues and tumor tissues with qRT-PCR. **b** The expression of miR-409-3p was detected in normal cells and tumor cells with qRT-PCR. **c** The expression of miR-409-3p was measured in inhibitor NC and miR-409-3p inhibitor groups with qRT-PCR. **d** The expression of miR-409-3p was measured in si-NC, si-circ_0000285, si-circ_0000285 + inhibitor NC and si-circ_0000285 + miR-409-3p inhibitor groups with qRT-PCR. **e** The expression of IGFBP3 was detected in normal tissues and tumor tissues with western blot. **f** The expression of IGFBP3 was detected in normal cells and tumor cells with western blot. **g** The expression of IGFBP3 was detected in si-NC and si-IGFBP3 groups with western blot. **h** The expression of IGFBP3 was detected in inhibitor NC, miR-409-3p inhibitor, miR-409-3p inhibitor + si-NC and miR-409-3p inhibitor + si-IGFBP3 groups with western blot. **i** The expression of IGFBP3 was detected in si-NC, si-circ_0000285, si-circ_0000285 + pc-NC and si-circ_0000285 + pc-IGFBP3 groups with western blot. **j** The expression of IGFBP3 was detected in si-NC, si-circ_0000285, si-circ_0000285 + inhibitor NC and si-circ_0000285 + miR-409-3p inhibitor groups with western blot. *P < 0.05

To further investigate the effect of miR-409-3p and IGFBP3 on OS, we found that miR-409-3p expression was decreased (Fig. 4a, b) while IGFBP3 expression was increased (Fig. 4e, f) in OS tissues and cells. Transfection of miR-409-3p inhibitor specifically reduces the expression of miR-409-3p (Fig. 4c). Besides, knockdown of circ_0000285 significantly upregulated the expression of miR-409-3p, while this upregulation effect was reversed when co-transfected si-circ_0000285 and miR-409-3p inhibitor (Fig. 4d). Moreover, the level of IGFBP3 protein was decreased when OS cells transfected with si-IGFBP3 (Fig. 4g), and it was increased by inhibiting the expression of miR-409-3p, although the co-transfection of si-IGFBP3 and miR-409-3p inhibitor would reverse the promotion effect induced by miR-409-3p inhibitor (Fig. 4h). Subsequently, we detected the relationship between circ_0000285 and IGFBP3. As shown in Fig. 4i, j, the protein level of IGFBP3 was positively associated with circ_0000285, overexpression of IGFBP3 or inhibition of miR-409-3p would antagonize the downregulated effect caused by si-circ_0000285.

### Circ_0000285/miR-409-3p/IGFBP3 axis was associated with OS progression

**Fig. 5:**
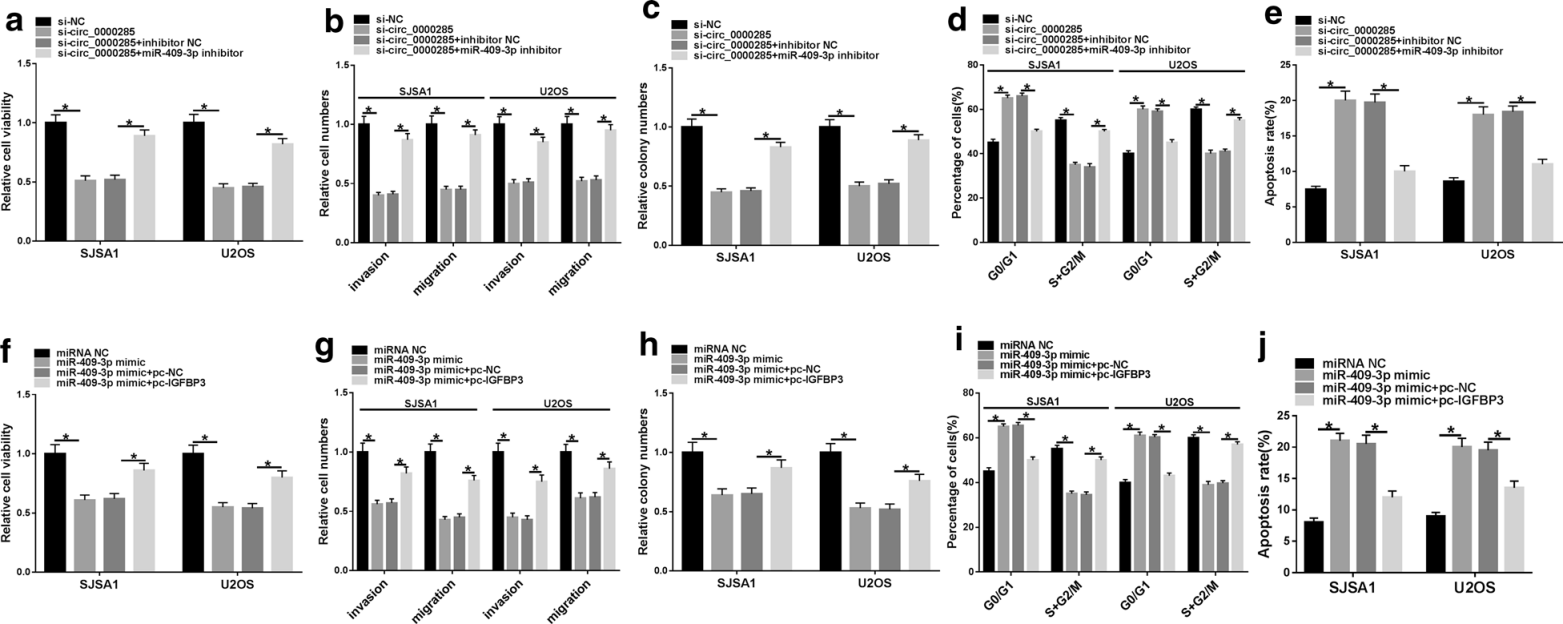
Circ_0000285/miR-409-3p/IGFBP3 axis was associated with OS progression. **a**–**e** Cell viability, migration, invasion, colony numbers, cycle and apoptosis were detected in si-NC, si-circ_0000285, si-circ_0000285 + inhibitor NC and si-circ_0000285 + miR-409-3p inhibitor groups. **f**–**j** Cell viability, migration, invasion, colony numbers, cycle and apoptosis were detected in miRNA NC, miR-409-3p mimic, miR-409-3p mimic + pc-NC and miR-409-3p mimic + pc-IGFBP3 groups. *P < 0.05

To further explore the function of circ_0000285/miR-409-3p/IGFBP3 axis in OS, rescue experiments were applied in SJSA1 and U2OS cells. We found that inhibition of circ_0000285 weakened cell viability, invasion, migration and colony numbers of cells while promoted cell apoptosis in SJSA1 and U2OS cells, which was reversed by suppressing miR-409-3p expression (Fig. 5a–e). Not only that, the effect of high miR-409-3p expression on the proliferation, invasion, migration, colony numbers and apoptosis of SJSA1 and U2OS cells was also similar to that of low circ_0000285 expression. However, increasing IGFBP3 expression could alleviate the inhibitory effect of miR-409-3p mimic on cell progression of SJSA1 and U2OS cells (Fig. 5f–j). Therefore, circ_0000285 regulated cell progression through miR-409-3p/IGFBP3 axis in OS.

### Knockdown of circ_0000285 suppressed tumor growth in OS

**Fig. 6:**
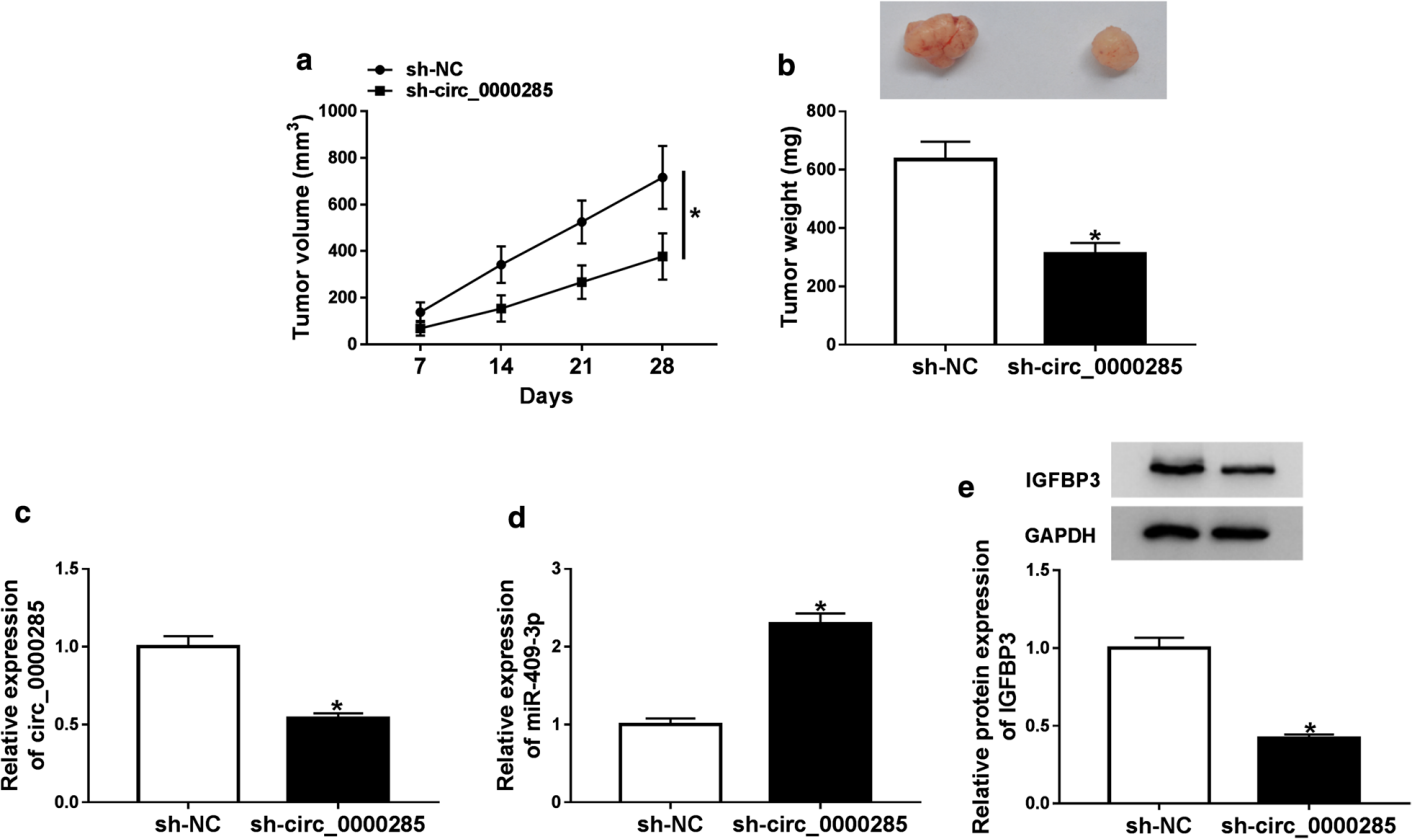
Knockdown of circ_0000285 suppressed tumor growth in OS. **a**, **b** Tumor volume and weight was measured in sh-circ_0000285 groups and sh-NC groups. **c**, **d** The expression of circ_0000285 and miR-187-3p was measured in sh-circ_0000285 groups and sh-NC groups with qRT-PCR. **e** The protein expression of IGFBP3 was detected in sh-circ_0000285 groups and sh-NC groups with western blot. *P < 0.05

To further explore the function of circ_0000285 in OS in vivo, we injected sh-NC and sh-circ_0000285 transfected cells subcutaneously into mice, and tumor width and tumor length were measured every 7 days. At 28 days of in vivo transfection, mice were killed and tumor weight was measured. The results showed that the growth curve of the tumor volume of sh-circ_0000285 was significantly lower than that of sh-NC (Fig. 6a). In addition, inhibition of circ_000024 expression could significantly reduce tumor weight (Fig. 6b). Moreover, sh-circ_0000285 transfection could decrease the expression of circ_0000285, increased the expression of miR-409-3p and reduced the protein expression of IGFBP3 in OS progression (Fig. 6c–e). These results indicated that inhibition of circ_0000285 significantly decreased tumor growth in OS.

## Discussion

At present, the etiology and pathogenesis of OS are still unclear. The abnormal proliferation of OS cells might be caused by the activation of proto-oncogenes, thereby affected cell proliferation, growth and replication [2, 21]. CircRNA has been shown to play a very important regulatory role in cancer development [22–25]. For example, circ_0001649, as a tumor suppressor, was down-regulated in pancreatic ductal adenocarcinoma tissues and affected tumor growth and differentiation [26]. Besides, circ_SMARCA5, acted as a tumor promoter, was up-regulated in prostate cancer tissues and cells and was related to cell proliferation and apoptosis [27]. In this article, hsa_circ_0000285 (circ_0000285) was found to be highly expressed in OS. Recent studies have shown that circ_0000285 could regulate the capacities of proliferation and migration and circ_0000285 overexpression enhanced the proliferative and migratory potentials of OS [28, 29]. In the study, inhibition of the expression of circ_0000285 can inhibit the growth of OS cells and promote their apoptosis. Therefore, circ_0000285 acted as an oncogene in OS consistent with a previous study [28] and was involved in cell progression in OS.

Next, we found that miR-409-3p was a target of circ_0000285 in OS. Various evidences indicated that miR-409-3p was more important in the physiological mechanisms of cancer, such as tongue squamous cell carcinoma, OS, non-small cell lung cancer, colon cancer, breast cancer, etcetera [30–34]. For example, miR-409-3p was downregulated in breast cancer and inhibited cellular process and invasion. More than that, miR-409-3p, as a marker, was associated with prognosis of patients with breast cancer [34, 35]. Similar to previous studies, miR-409-3p was downregulated in OS tissues and cells [31, 36]. Reverse experiments indicated that circ_0000285 targeted miR-409-3p to regulate cell proliferation, apoptosis, migration and invasion. Therefore, miR-409-3p has been verified to play a key role in cell progression of OS.

Then, dual-luciferase reporter assay also determined that IGFBP3 was a target mRNA of miR-409-3p in OS. Interestingly, previous studies determined that IGFBP3 was a target mRNA of miR-196b, miR-1290, miR-384, miR-34/449 and miR-155 in cancers [37–40], but the regulatory mechanism of miR-409-3p/IGFBP3 has not been verified in OS. Accumulating researches indicated that IGFBP3 played an important role in regulating cell cycle and apoptosis in various cancers, including breast cancer, nasopharyngeal carcinoma, OS, etcetera [41–44]. For example, IGFBP3 expression was increased in nasopharyngeal carcinoma and IGFBP3 was also associated with cell migration and adhesion [42]. In OS, IGFBP3 was up-regulated in OS and involved in cell proliferation and invasion of OS [38]. In our study, IGFBP3 expression was induced in OS tissues and cells. Overexpression of IGFBP3 could reverse the suppressive effects of high miR-409-3p expression on cell progression in OS. Thus, miR-409-3p targeted IGFBP3 to regulate OS cell proliferation and apoptosis. More than that, we also verified that circ_0000285 directly targeted miR-409-3p to regulate IGFBP3 expression in OS.

## Conclusion

In conclusion, we for the first time identified and verified that circ_0000285 affected the cellular progression of OS via directly targeting miR-409-3p that regulated IGFBP3 expression, providing a new biomarker for the treatment of OS and a novel regulation mechanism for the pathogenesis of OS.

## Supplementary information

**Additional file 1:** **Table S1.** Correlation between circ_0000285 expression and clinical clinicopathological parameters of OS.

## Data Availability

The analyzed data sets generated during the present study are available from the corresponding author on reasonable request.
