# Supplementary material for: Circ_0000285 regulates proliferation, migration, invasion and apoptosis of osteosarcoma by miR-409-3p/IGFBP3 axis
Source: Cancer Cell Int. 2020 Oct 6;20:481. doi: 10.1186/s12935-020-01557-5 (PMC7539413; doi:10.1186/s12935-020-01557-5)
Supplement: Supplementary file 1 — Additional file 1: Table S1. Correlation between circ_0000285 expression and clinical clinicopathological parameters of OS. [file 12935_2020_1557_MOESM1_ESM.docx]

Table 1. Correlation between circ_0000285 expression and clinical

clinicopathological parameters of OS

| Parameter | Case | circ_0000285 expression | | *P* value^a^ |
| --- | --- | --- | --- | --- |
|  |  | Low(n=16) | High(n=14) |  |
| Age (years) |  |  |  | 0.143 |
| ≤25 | 15 | 6 | 9 |  |
| >25 | 15 | 10 | 5 |  |
| Gender |  |  |  | 0.765 |
| Female | 18 | 10 | 8 |  |
| Male | 12 | 6 | 6 |  |
| Tumor size |  |  |  | 0.001* |
| ≤5 cm | 16 | 13 | 3 |  |
| >5 cm | 14 | 3 | 11 |  |
| WHO grade |  |  |  | 0.431 |
| I-II | 13 | 8 | 5 |  |
| III | 17 | 8 | 9 |  |
| Pulmonary metastasis |  |  |  | 0.282 |
| YES | 14 | 6 | 8 |  |
| NO | 16 | 10 | 6 |  |
| Differentiation grade |  |  |  | 0.052 |
| Well/moderately | 12 | 9 | 3 |  |
| Poorly/undifferentiated | 18 | 7 | 11 |  |

OS: Osteosarcoma; WHO: World Health Organization; **P* < 0.05 ^a^Chi-square test
